# Comparative Recurrence Analysis of Pancreatic Adenocarcinoma after Resection

**DOI:** 10.1155/2021/3809095

**Published:** 2021-10-21

**Authors:** Chaobin He, Zhiyuan Cai, Yu Zhang, Xiaojun Lin

**Affiliations:** ^1^Department of Pancreatobiliary Surgery, State Key Laboratory of Oncology in South China, Collaborative Innovation Center for Cancer Medicine, Sun Yat-Sen University Cancer Center, Guangzhou 510060, China; ^2^Guangdong Provincial Engineering Research Center of Molecular Imaging, The Fifth Affiliated Hospital of Sun Yat-Sen University, Zhuhai 519000, China; ^3^State Key Laboratory of Ophthalmology, Zhongshan Ophthalmic Center, Sun Yat-Sen University, Guangzhou 510060, Guangdong, China

## Abstract

**Purpose:**

The relation between tumor sites of pancreatic ductal adenocarcinoma (PDAC) and recurrence was not fully investigated before. We aimed to describe the differences of recurrent patterns in PDAC of head and body/tail after curative surgery.

**Methods:**

The recurrent patterns of PDAC were compared and the associations with clinical characteristics were analyzed in these patients. Prognostic factors of overall survival (OS) and progression-free survival (PFS) were analyzed and validated. Predictive systems were constructed and measured by the area under the AUC curve and concordance index (C-index).

**Results:**

A total of 302 PDAC patients were included in this study, including 247 patients with PDAC of head and another 55 patients with PDAC of body/tail. Patients who developed tumor recurrence within 24 months after resection had significantly shorter OS in both groups. Liver metastasis occupied most of the tumor progressions and diminished while local recurrence increased gradually over time. The variation trends were similar for patients in both groups while these changes were more pronounced for patients in the head group. Local recurrence and liver-only metastasis seemed to indicate a better OS. Furthermore, predictive systems for OS and PFS prediction based on independent risk factors were established and showed significant higher values of AUC and C-indexes compared with the TNM stage system.

**Conclusions:**

Different characteristics of progressions for PDAC of head and body/tail suggested biological heterogeneity. The exploration of these variations helps to provide personalized management of recurrence in PDAC.

## 1. Introduction

As a lethal disease with increasing morbidity, pancreatic ductal adenocarcinoma (PDAC) is predicted to cause the second most number of cancer-specific deaths by 2030 [1]. Surgery provides the best chance to obtain prolonged survival while this option is eligible for only 20% of all PDAC patients [2]. The late diagnosis, rapid tumor progression, and early tumor recurrence after treatment contributed to the high inoperability and poor prognosis of PDAC [3, 4]. Although treatment strategies have been improving all along, most patients relapse and succumb to this disease. After surgery, up to 80% of patients suffered from early recurrence [5, 6] and the 5-year survival rate was less than 10% [7].

Different sites of tumors were shown to have different characteristics [8, 9], indicating that tumor locations may affect carcinogenesis in a tissue greatly. In terms of PDAC, the discrepancies of ontogeny would lead to great variations in cell composition and blood supply in PDAC of the head and body/tail [10]. Because of the absence of specific symptoms, PDAC in the body/tail of pancreas is generally larger and more likely to develop metastases at diagnosis [11]. Besides, more aggressive tumor biology was indicated in PDAC of the body/tail [12]. These differences may greatly impact recurrent patterns between PDAC of head and body/tail. Similarly, previous studies have shown that multiple anatomic sites of PDAC may contribute to the varied survival of patients [13, 14]. However, the relations between primary tumor site and recurrence timing and patterns of PDAC have not been investigated yet. Considering the close relationship between prognosis and progression in PDAC [4], exploration of the differences in risk factors, timing, and patterns of progressions can help personalized treatment.

## 2. Patients and Methods

### 2.1. Patients

As a continuous study of our previous research, the inclusion and exclusion criteria were reported before [4]. Briefly, all patients who were pathologically confirmed PDAC and had received radical resection from 2008 to 2018 at Sun Yat-sen University Cancer Center (SYSUCC) were retrospectively included in this study. Excluded patients were those with metastatic diseases detected at diagnosis by radiological examination. Those with microscopic or macroscopic incomplete resection or missing follow-up information were also excluded from this study. The resection margin for radical resection was defined as 1.5–2 mm, which was the same as previous studies [4, 15, 16]. This study was conducted in accordance with the ethical standards of Helsinki Declaration and was approved by the Institutional Review Board of SYSUCC.

### 2.2. Data Collection

All included patients had received radical resection and the pathological diagnosis of PDAC was finished by an experienced pancreatic pathologist. The following pathological factors were analyzed, including tumor size, differentiation, lymph node (LN) metastasis, LN total and positive number, satellite foci, vascular, lymph vessel, perineural and adjacent organ invasion, and combined venous resection. Lymph node ratio (LNR) is defined as the ratio between the number of positive LNs and the total number of examined LNs. In addition, the associated radiological and clinical variables, which had been described in our previous studies [4], were collected within 7 days before surgery in this study [4].

### 2.3. Recurrence Patterns

Information on recurrence patterns was obtained through strict follow-up after surgery. Either radiological or histological evidence was required for the diagnosis of recurrence of disease. The specific recurrence pattern was defined as the first location of recurrence. Similar with the study of Groot et al. [6], five categories were included. The “Liver-only,” “Lung-only,” and “Others” metastases referred to the isolated hepatic, pulmonary recurrence, and isolated recurrence in other less common areas, respectively. In addition, “Local + distant” or “Multiple” metastases referred to local recurrence, and isolated distant metastasis happened simultaneously or as multiple distant metastases, respectively.

### 2.4. Survival Outcomes and Statistical Analysis

The follow-up of patients occurred at the outpatient clinic of our hospital. In general, follow-up strategies consisted of regular chest computed tomography (CT), abdominal CT, and CA19-9 test, at least every 2 months during the first year after surgical resection and every 3 months thereafter. Occasional additional imaging modalities, such as magnetic resonance imaging (MRI) and positron emission tomography/CT (PET/CT), were selectively performed to determine patterns of recurrence. Patients who had LN metastases or other risk factors, including macrovascular or microvascular invasion, and lymph vessel invasion, were recommended to receive chemotherapy. Two survival outcomes were analyzed in this study, including progression-free survival (PFS) and overall survival (OS), defined as the time from surgery to progression and death, respectively, or last follow-up. In addition, post-progression survival (PPS), defined as the time from first tumor progression to death or last follow-up, was also evaluated in this study. The date of the last follow-up was at the end of May 2019. Kaplan–Meier method was used to estimate survival and the differences of survival were compared with the log-rank test. Factors that were statistically significant in the univariable analysis and least absolute shrinkage and selection operator (LASSO) logistic regression were candidates for entry into a multivariable analysis. Area under the receiver operating characteristic (ROC) curves (AUC) and concordance index (C-index) of the multimarker algorithms were calculated to compare the predictive efficacy of risk factors with that of the tumor-node-metastasis (TNM) stage system. All *P* values were two-sided and *P* values < 0.05 were considered significant. *R* software version 3.6.1 (R Development Core Team; http://www.r-project.org) was used to conduct all statistical analyses.

## 3. Results

### 3.1. Patients

Between 2008 and 2018, 355 patients underwent surgical resection and were histologically confirmed PDAC at SYSUCC. A total of 53 patients who did not meet the criteria for inclusion were excluded from this study: 10 patients with microscopic or macroscopic incomplete resection, 12 patients with second primary tumors, and 31 patients with incomplete follow-up information. Finally, there were a total of 273 patients who were diagnosed with resectable diseases and another 29 patients diagnosed with borderline resectable diseases. All patients have received radical resection (R0 resection). All patients were followed up for more than 1 year and the median follow-up time was 24.7 months [95% confidence interval (CI) 20.3–29.1] after surgery. Tumor recurrence was detected in a total of 173 (57.3%) patients while there was no recurrence in 129 (42.7%) patients. For patients with and without recurrence, the median follow-up time was 13.8 and 40.6 months, respectively (Table 1).

### 3.2. Timing of Recurrence

According to the primary tumor sites, patients were sorted into the head and body/tail groups, respectively. There were 247 patients in the head group and another 55 patients in the body/tail group. A total of 140 and 24 patients in the head and body/tail groups were younger than 60 years, respectively. Male patients accounted for 40% of all patients in both groups. The median values of tumor size were 3.5 cm (range 1.0–8.9) and 3.9 cm (range 2–10) in the head and body/tail group, respectively. The mean number of LN retrieved is 12.89 and the median value is 12. Similar ratios of LN metastasis were observed in both groups.

**Figure 1:**
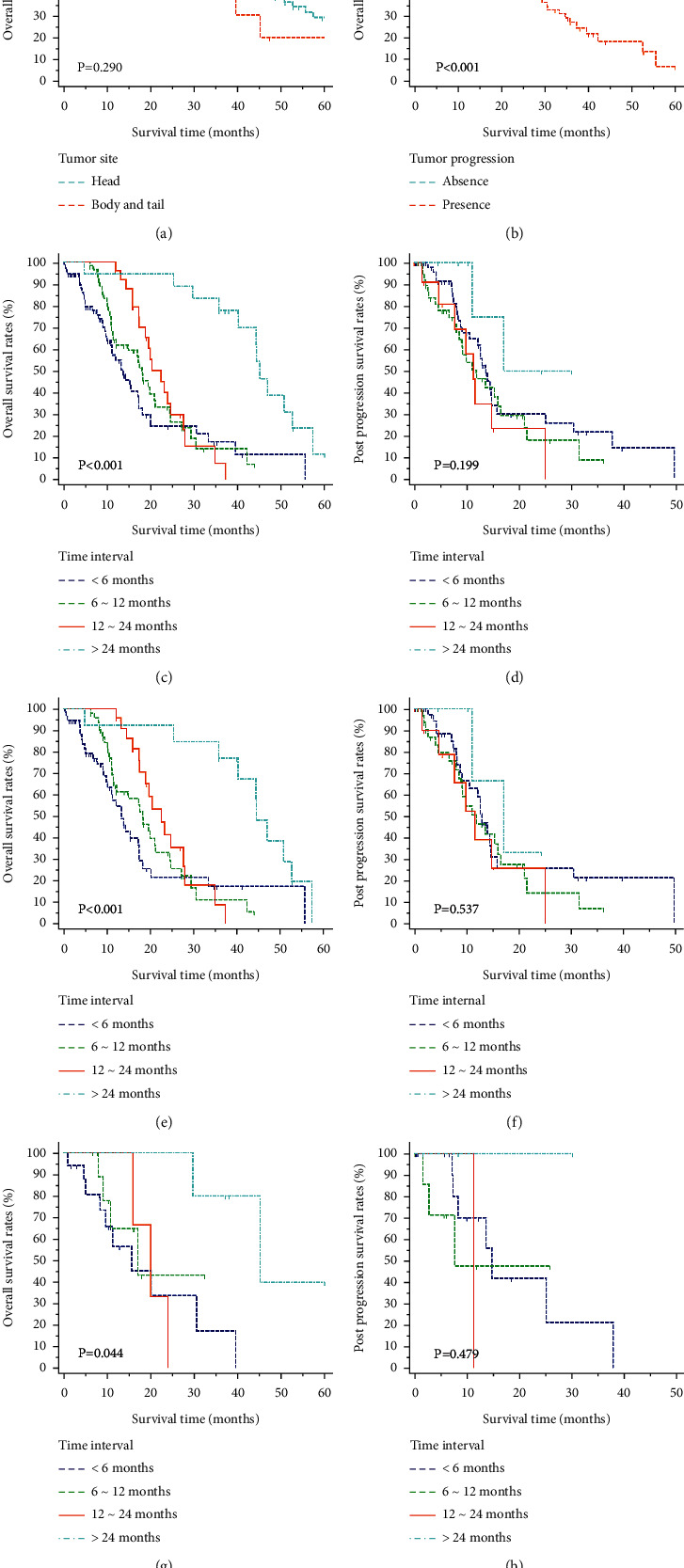
Overall survival (OS) and post-progression survival (PPS) analysis for PDAC patients. OS stratified by tumor site (a), tumor progression (b), and time period to tumor progression (c). PPS stratified by time period to tumor progression (d) in all PDAC patients. OS and PPS stratified by time period to tumor progression in PDAC patients of the head (e, f) and body/tail (g, h).

Overall, among 173 patients who had developed recurrences, most patients had done so within 24 months. Patients with tumor progressions had significantly shorter survival than those without recurrences. In terms of survival comparisons, patients in the head group seemed to have longer OS while the survival differences were not significant (Figure 1). It was shown that patients who developed recurrence within 24 months had significantly shorter OS than those beyond 24 months, while PPS did not differ significantly between these two groups. In addition, patients had similar OS and PPS when they developed recurrences within 6, 6–12, or 12–24 months after surgery. Similar results were also obtained in PDAC patients of both groups (Figure 1).

### 3.3. Patterns of Recurrence

**Figure 2:**
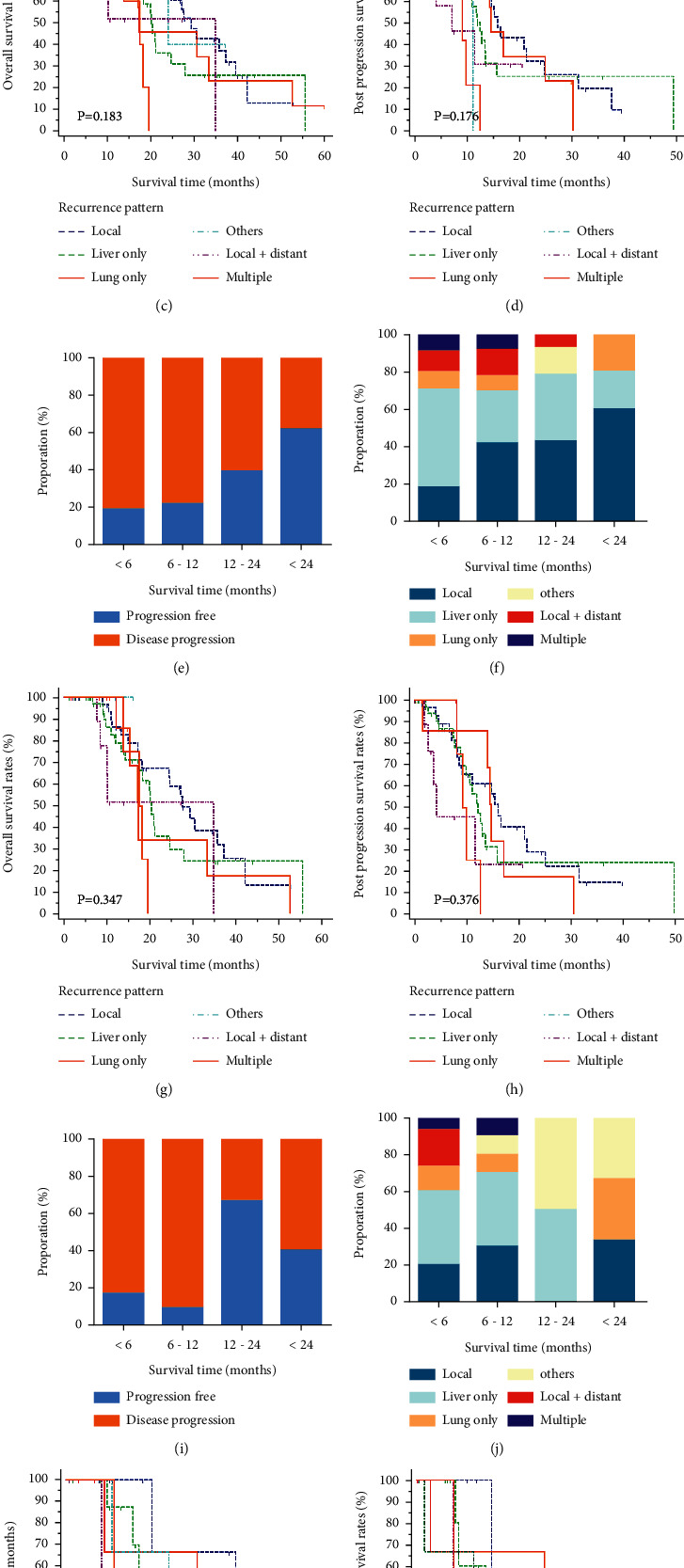
Distribution of tumor progression patterns at different time points and their survival analyses. The proportions of tumor progression patterns (a, b). The OS (c) and PPS (d) stratified by tumor progression patterns in all PDAC patients. The proportions of tumor progression patterns (e, f). The OS (g) and PPS (h) stratified by tumor progression patterns in PDAC patients of the head. The proportions of tumor progression patterns (i, j). The OS (k) and PPS (l) stratified by tumor progression patterns in PDAC patients of the body/tail.

**Figure 3:**
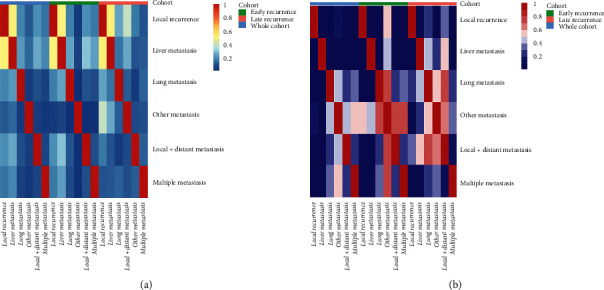
The heat maps of correlation coefficient (a) and the associated *P* values (b) of tumor progression patterns. The development of liver-only metastasis showed significantly negative relations with other kinds of progression patterns and these relationships were more obvious in the early progression group (earlier than 1 year since surgery) than those in the late progression group (later than 1 year since surgery) among the whole, head, and body/tail groups.

A total of six types of recurrence were recorded for progression. Liver metastasis occupied most of the tumor progression types, followed by local progression, local and distant progression, and lung metastasis. Metastases in other sites or multiple metastases contributed to only a small part of all tumor progressions. Similar proportions of recurrence patterns were observed in both groups. The comparisons of distributions for these recurrence patterns in the whole, head, and body/tail groups are shown in Figure 2. The proportions of tumor progression seemed to decrease over time and most progressions happened within one year after surgery. In addition, this descend range was more obvious in patients of the head group, compared with those in the body/tail group. In terms of specific recurrence pattern, it was shown that within 6 months after surgery, liver-only metastasis was the major form of tumor progression. As time went on, the proportions of liver-only metastasis decreased gradually while local recurrence and lung-only metastasis contributed to more and more progressions (*P* < 0.001). This trend could be observed in the whole, head, and body/tail groups, and it was more obvious in patients of the head group. In addition, these changes could also be reflected in the correlations of different patterns of recurrences, which are shown in Figure 3. The development of liver-only metastasis showed significantly negative relations with other kinds of progression patterns and these relationships were more obvious in the early progression group (earlier than 1 year since surgery) than those in the late progression group (later than 1 year since surgery) among patients in the whole, head, and body/tail groups.

Varied progression patterns contributed to different cumulative survival rates. It was indicated that patients with multiple metastases shared significantly shorter OS and PPS than those with other types of progression patterns, whereas the survival rates of local, lung only, liver only, other sites, and local plus distant metastases were similar in patients of the head and body/tail groups (Table 2). The pairwise comparisons of OS and PPS for different types of progression patterns were also conducted. Local recurrence and liver-only metastasis seemed to indicate a better OS while patients with local recurrence and lung-only metastasis obtained a little longer PPS than those with other types of tumor progressions. However, these survival benefits were not significant for patients with PDAC in the head and body/tail groups.

### 3.4. Risk Factors for OS and PFS

For patients in the head group, the 1-, 2-, and 3-year OS and PFS were 81.7%, 59.9%, and 48.3%, and 51.7%, 37.5%, and 33.2%, respectively. Similarly, the 1-, 2-, and 3-year OS and PPS were 76.1%, 50.7%, and 40.6%, and 31.4%, 24.4%, and 9.3%, respectively, for patients in the body/tail group. Although no significant variations in OS for patients in the head and body/tail groups were observed, those in the head group had significantly longer PFS, compared with patients in the body/tail group (*P*=0.002).

**Figure 4:**
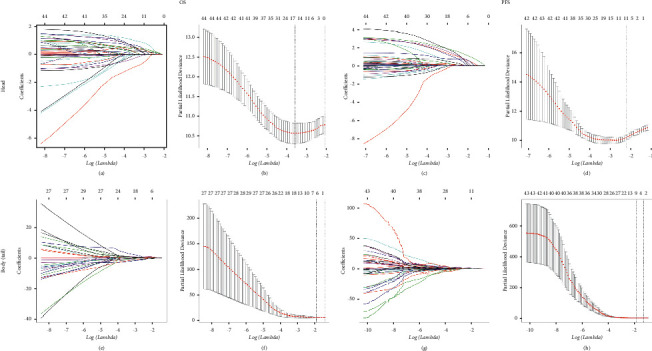
Feature selection using the least absolute shrinkage and selection operator (LASSO) cox regression model. LASSO coefficient profiles of 48 variables against the log (Lambda) sequence and tuning parameter selection in the LASSO model used 10-fold cross-validation via minimum criteria for survival (PDAC of the head, OS (a, b), and PFS (c, d); PDAC of the body/tail, OS (e, f), and PFS (g, h)).

LASSO regression was conducted based on 48 high-dimensional radiological and pathological data to investigate the prognostic factors (Figure 4). Seven variables were selected for OS prediction in both groups, including local progression, liver-only or lung-only metastasis, local plus distant recurrences, tumor differentiation, LN16 metastasis, and imaging tumor size. In terms of PFS prediction, the selected predictors were TNM stage, local progression, liver-only metastasis, lung-only metastasis, local plus distant recurrences, multiple recurrences, LN16 metastasis, invasion of back membrane in pancreas, imaging tumor size, number of positive LN, and LNR for patients in the head group, and pathological tumor size, imaging vascular invasion, and imaging LN size for patients in the body/tail group.

Factors that were positive in the LASSO regression and univariable analysis were included and analyzed in the multivariable analysis. It was illustrated that decreased time interval to progression (HR = 18.34, 95% CI 7.00–48.05, *P* < 0.001), LN16 metastasis (HR = 2.51, 95% CI 1.02–6.17, *P*=0.046), tumor differentiation (HR = 3.52, 95% CI 1.45–5.31, *P*=0.002), local progression (HR = 7.09, 95% CI 3.65–13.90, *P* < 0.001), liver-only metastasis (HR = 11.49, 95% CI 5.35–24.40, *P* < 0.001), lung-only metastasis (HR = 4.78, 95% CI 1.87–12.35, *P*=0.010), and local plus distant recurrence (HR = 4.21, 95% CI 1.14–15.55, *P*=0.031) were independent predictors for reduced OS (Table 3). Moreover, CEA (HR = 1.79, 95% CI 1.17–2.73, *P*=0.007), chemotherapy (HR = 0.48, 95% CI 0.30–0.75, *P*=0.001), imaging tumor size (HR = 1.703, 95% CI 1.20–3.65, *P*=0.029), local progression (HR = 13.64, 95% CI 7.28–25.57, *P* < 0.001), liver-only metastasis (HR = 18.63, 95% CI 10.51–33.04, *P* < 0.001), lung-only metastasis (HR = 19.31, 95% CI 7.05–52.88, *P* < 0.001), local plus distant recurrence (HR = 13.54, 95% CI 5.91–31.02, *P* < 0.001), multiple metastases (HR = 33.96, 95% CI 13.14–87.81, *P* < 0.001), and TNM stage (HR = 4.40, 95% CI 1.54–12.60, *P*=0.006) were identified as independent predictors for PFS for patients in the head group (Table 4). As for PDAC of the body/tail, decreased time interval to progression, local progression, liver-only metastasis, and tumor differentiation were identified as independent predictors for OS. In addition, it was shown that NLR, mGPS, pathological tumor size, and imaging LN size were able to predict PFS for PDAC of the body/tail. In terms of surgery-related complications, no significant relationships with OS and PFS were observed.

### 3.5. Performance of Prediction for OS and PFS

The predictive power of significant predictive factors was further validated. It was indicated that the values of AUC for 1-, 2- and 3-year OS and PFS prediction were 0.720, 0.734, and 0.801, and 0.749, 0.749, and 0.748, respectively, for patients in the head group. It was shown that compared with the 8^th^ TNM stage system, higher values of AUC for the predictive factors were observed. Moreover, significantly higher values of C-indexes were also observed for OS (0.688, 95% CI 0.623–0.753) and PFS (0.800, 95% CI 0.760–0.840) for PDAC of head (both *P* < 0.050). In terms of PDAC in the body/tail group, the selected predictive factors also exhibited significantly higher values of AUC and C-indexes compared with the 8^th^ TNM stage system (Table 5).

## 4. Discussion

As the main reason for poor prognosis, tumor recurrence is the major reason for PDAC after surgery. Similar to those from other studies [6, 17], it was observed that 57.3% of patients had developed recurrence which would lead to significantly poorer survival. Most progressions occurred within 2 years at distant sites, suggesting that PDAC was a systemic disease at the time of surgery. Therefore, it is important to explore the timing and patterns of PDAC after surgery. Considering the differences of tumor origin, the characteristics and survival impact of recurrences in PDAC of head and body/tail may be different. This study compared the timing and patterns of recurrences and investigated the relation between recurrence characteristics and survival in PDAC in the head and body/tail groups for the first time. The analysis of recurrence timing and patterns, which are two important aspects of tumor progression, may help to explore the unique biological behaviors of PDAC. (Table 5)

Similar with timing distribution of progression in all patients, tumor progression occurred mainly in the first two years after surgery and this was a linear trend of decrease in recurrence probabilities over time for patients with PDAC of head and body/tail. Around 10.4% of progressions could also be observed 2 years after surgery, showing that recurrence-free survival for two years did not mean cure for PDAC. In addition, the recurrence rate was even higher in PDAC of body/tail. Compared with tumors in the head, those in the body/tail was more likely to progress at two years after surgery. This could be due to the late onset of symptoms of body/tail, leading to more finding of recurrence in two years after surgery.

Further analysis for the distribution of progression patterns in PDAC was also conducted. Liver-only metastasis and local recurrence contributed to most of disease progressions for PDAC in the both groups. In addition, when time period to metastasis was considered, it was shown that local recurrence increased gradually and represented a majority of tumor progression forms in two years after surgery. On the contrary, most of liver-only metastasis occurred in the first two years after surgery and diminished over time. This trend for liver-only metastasis was more obvious for all PDACs and PDAC in the head group, compared with those in the body/tail group. Significantly, negative correlations were also observed between liver-only metastasis and other types of tumor progression. Apart from local recurrence, the ratios of lung-only metastasis also increased along with time and PDAC of body/tail was more likely to develop lung-only metastasis compared with PDAC of head. In terms of local plus distant metastasis and multiple metastases, they were mostly observed in early period after surgery in small groups of patients. Considering the changes of progression patterns over time, patients could benefit from the changes of treatment focus during the periods of follow-up for PDAC.

Apart from the varied distributions of timing and patterns of tumor progressions, there were also survival differences among different timing and patterns of progressions. Among all types of tumor progression, local recurrence had the longest OS of 29.37 months in the whole groups of patients and 27.6 months in the head group, respectively, followed by other and lung-only metastases. With regard to tumor progression, similar with other studies [5], liver-only metastasis contributed to the shortest PFS, which was similar with that for PDAC with local + distant and multiple metastases. Considering the high prevalence of liver metastasis, which may lead to most of local + distant and multiple metastases, it was reasonable for the similarities of survival rates among these progression types. Although liver-metastasis had the poorest PFS, its median PPS was as long as 14.7 months and was only shorter than that of local recurrence. Apart from local recurrence, patients with lung or other metastases also had relatively long PFS or PPS, respectively, which contributed to significantly longer OS than that of patients with liver-only, local + distant and multiple metastases. Compared with liver or lung metastases, a larger tumor bed and the functional preservation of other metastases were necessary for obtaining longer survival [18]. These survival results were consistent among all PDAC patients and those in the head and body/tail groups. In addition, considering the less aggressive nature and slow growth pattern of local regression and lung-only metastasis, additional treatment could also provide some space for survival elevation in patients with subsequent lung-only metastasis or local recurrence.

In the further analysis of the impact of radiological and pathological factors on OS and PFS, it was shown that PDAC in both groups shared most of the risk factors, including time period to progression, tumor differentiation, local progression, and liver-only metastasis. Apart from these risk factors, LN16, lung-only, and local plus distant metastases also indicated significantly poorer survival for PDAC patients in the head group. In addition, the prognostic factors of PFS were also explored in this study. It was indicated that CEA, chemotherapy, local regression, liver-, lung-, local, and distant metastases were independent factors of PFS for PDAC in the head group, while NLR, mGPS, pathological tumor size, and LN size predicted PFS in PDAC in the body/tail group. Decreased time to progression, which reflected a more malignant nature of the disease, indicated poorer survival in both head and body/tail groups, and it was more obvious for the latter. Similar to our study, a pool study of 692 PDAC patients also showed decreased survival due to the decreased time to tumor progression [6]. Besides, poorly differentiated tumor also indicated poor OS in patients. It was shown that epidermal growth factor and E-cadherin could be released by poorly differentiated tumors, enhancing the ability to develop distant metastases [19]. In terms of the recurrence patterns, survival of PDAC in the body/tail group was more likely to be affected by local recurrence and liver metastases, which acted as the main forms of disease progression, while the prognosis of PDAC in the head group could be influenced by multiple types of disease progressions. Elevated level of CEA and increased size of tumor or metastatic LNs were significantly associated with poor survival, indicating that PDAC patients with these unfavourable characteristics may need to receive more strict follow-up strategies and additional specific therapy to prolong survival. Consistent with the results from the study by Groot [20], our results also illustrated that chemotherapy was helpful for increasing PFS for PDAC patients in both groups. The elimination of potential disease by chemotherapy might contribute to prolonging survival after surgery. However, chemotherapy was not shown as an independent predictor for OS. Controversial results concerning chemotherapy on OS of PDAC were observed and the variations of length and regimens of chemotherapy, along with the selection biases, could potentially lead to these conflicting results [21, 22]. Probably, more insights concerning survival benefit from uniformed regimens and periods of chemotherapy in prospective studies are needed.

The predictive systems for OS and PFS prediction were established in this study. Additional independent risk factors were included in the predictive systems, guaranteeing the enhanced strength of the predictive system, compared with the TNM system. On the other hand, the differences of prognosis for PDAC in both groups indicated that probably individual predictive system was needed for these two kinds of diseases, which was reflected by the variations of predictive factors specially designed for PDAC of head and body/tail, respectively. It is well-known that precise prediction of survival is essential for individual treatment. Clinicians can perform evaluation of survival rates based on these independent risk factors and specialize in the adjuvant therapies, which are helpful for personalized medicine.

There were several limitations to this study. First, some variables, including specific treatment after surgery, the time period and regimen of chemotherapy, were still unavailable for this study. The inclusion of these variables would further improve the feasibility of the predictive system of survival for PDAC. Second, only the first recurrence was recorded in this study. Third, tumor progressions would be greatly affected by the length of the follow-up period. A longer time period of follow-up was also needed for a more precise overview of tumor progression after surgery. Finally, further validation based on prospective cohorts with more patients was needed for the present study.

## 5. Conclusions

In conclusion, the comparisons of the timing and patterns of recurrences and investigation of the relations between recurrence characteristics and survival in PDAC of the head and body/tail were conducted in this study for the first time. It was shown that there were some differences in the recurrence timing and patterns of progressions for PDAC of head and body/tail. The associated risk factors for OS and PFS were selected for these two kinds of diseases, respectively. Furthermore, specialized predictive systems were also established and were shown to exhibit great predictive power for survival prediction. The conduction of the predictive system would be greatly helpful for the personalized management for PDAC of head and body/tail after surgery.

## Figures and Tables

**Table 1:** Clinicopathological characteristics of patients with PDAC stratified by tumor site.

Characteristics		Tumor site				Characteristics		Tumor site			
		Head	Body/tail	N	*P*			Head	Body/tail	N	*P*
Whole cohort		247	55	302		Macrovascular invasion	Absence	231	42	273	<0.001
Age	≤60 years	140	24	164	0.099		Presence	16	13	29	
	>60 years	107	31	138		Microvascular invasion	Absence	162	44	206	0.039
Gender	Male	97	22	119	1.000		Presence	85	11	96	
	Female	150	33	183		Lymph vessel invasion	Absence	125	15	140	0.031
Recurrence	Absence	148	26	174	0.098		Presence	122	40	162	
	Presence	99	29	128		Perineural invasion	Absence	127	19	146	0.026
Time to recurrence	Absence	111	18	129	0.211		Presence	120	36	156	
	2–6 M	54	18	72		Adjacent organ invasion	Absence	247	23	270	<0.001
	6–12 M	46	11	57			Presence	0	32	32	
	12–24 M	23	3	26		LNR	0	135	38	173	0.140
	>24 M	13	5	18			0–0.16	58	8	66	
Recurrence patterns	Absence	148	26	174	0.157		>0.16	54	9	63	
	Local	32	7	39		Satellite foci	Absence	243	44	287	<0.001
	Liver-only	39	10	49			Presence	4	11	15	
	Lung-only	8	4	12		TNM stage	IA	49	5	54	<0.001
	Other sites	2	3	5			IB	64	10	74	
	Local + distant	11	3	14			IIA	22	13	35	
	Multiple	7	2	9			IIB	71	8	79	
LN metastasis	Absence	136	38	174	0.070		III	41	19	60	
	Presence	111	17	128		Imaging tumor size (cm)	≤2	97	7	104	<0.001
LN5 metastasis	Absence	245	55	300	1.000		2–4	123	18	141	
	Presence	2	0	2			>4	27	30	57	
LN6 metastasis	Absence	243	55	298	1.000	Imaging LN metastasis	Absence	133	42	175	0.002
	Presence	4	0	4			Presence	114	13	127	
LN7 metastasis	Absence	242	54	296	1.000	Imaging vascular invasion	Absence	209	25	234	<0.001
	Presence	5	1	6			Presence	38	30	68	
LN8 metastasis	Absence	241	53	294	0.641	Imaging LN size (cm)	≤0.5	139	38	177	0.216
	Presence	6	2	8			0.5–1	55	9	64	
LN9 metastasis	Absence	239	53	292	1.000		>1	53	8	61	
	Presence	8	2	10		PI	0	154	45	199	0.022
LN10 metastasis	Absence	247	48	295	<0.001		1	76	8	84	
	Presence	0	7	7			2	17	2	19	
LN11 metastasis	Absence	247	47	294	<0.001	NLR	≤3.32	153	44	197	0.012
	Presence	0	8	8			>3.32	94	11	105	
LN12 metastasis	Absence	213	55	268	0.001	dNLR	≤3.32	79	21	100	0.429
	Presence	34	0	34			>3.32	168	34	202	
LN13 metastasis	Absence	178	53	231	0.001	PLR	≤98.13	21	15	36	<0.001
	Presence	69	2	71			>98.13	226	40	266	
LN14 metastasis	Absence	227	54	281	0.141	PNI	0	54	11	65	0.857
	Presence	20	1	21			1	193	44	37	
LN15 metastasis	Absence	241	53	294	0.641	SII	≤1000	158	48	206	0.001
	Presence	6	2	8			>1000	89	7	96	
LN16 metastasis	Absence	231	53	284	0.544	mGPS	0	157	45	202	0.033
	Presence	16	2	18			1	60	7	67	
LN17 metastasis	Absence	238	55	293	0.373		2	30	3	33	
	Presence	9	0	9		WBC	≤10	227	53	280	0.389
LN18 metastasis	Absence	244	52	296	0.076		>10	20	2	22	
	Presence	3	3	6		ALB (g/L)	≤35	43	3	46	0.023
Positive LN number	0	135	38	173	0.142		>35	204	52	256	
	1–3	83	12	95		CRP (ng/L)	≤3	157	45	202	0.011
	>4	29	5	34			>3	90	10	100	
Pancreatic membrane invasion	Absence	163	20	183	<0.001	CA19-9 (U/ml)	≤35	49	10	59	0.853
	Presence	84	35	119			>35	198	45	243	
Tumor size (cm)	≤2	82	6	88	<0.001	CEA (ng/ml)	≤5	172	33	205	0.201
	2–4	125	21	146			>5	75	22	97	
	>4	40	28	68		HBV infection	Absence	229	54	283	0.216
Tumor differentiation	Well	0	2	2	0.010		Presence	18	1	19	
	Moderate	125	28	153		Chemotherapy	No	134	26	160	0.373
	Poor	122	25	147			Yes	113	29	142	
Hemorrhage	Absence	241	54	295	0.626	Biliary fistula	Absence	212	47	259	0.543
	Presence	6	1	7			Presence	35	8	43	
Pancreatic fistula	Absence	193	48	241	0.141	Abdominal infection	Absence	225	54	279	0.091
	Presence	54	7	61			Presence	22	1	23	

*M*, month; LN, lymph node metastasis; LNR, lymph node ratio; TNM, tumor-node-metastasis stage; PI, prognostic index; NLR, neutrophil-to-lymphocyte ratio; PLR, platelet-to-lymphocyte ratio; PNI, prognostic nutritional index; SII, systemic immune-inflammation index; mGPS, modified Glasgow Prognostic Score; WBC, white blood cell count; ALB, albumin; CRP, C-reactive protein; CA19-9, carbohydrate antigen 19–9; CEA, carcinoembryonic antigen; HBV, hepatitis B virus.

**Table 2:** Pairwise comparison of survival for different tumor progression patterns.

Recurrence patterns		Whole cohort									Head								
		OS			Aps			PFS			OS			Aps			PFS		
		Mst	95%CI	*P*	Mst	95%CI	*P*	Mst	95%CI	*P*	Mst	95%CI	*P*	Mst	95%CI	*P*	Mst	95%CI	*P*
Local progression	Reference	29.37	24.47–39.57		15.93	11.07–25.03		8.97	6.40–10.57		27.60	24.47–37.23		15.93	9.13–21.53		9.13	6.90–10.80	
	Liver-only metastasis	20.1	17.37–24.63	0.214	12.60	9.83–15.77	0.444	5.03	4.07–6.47	0.014	20.40	17.37–27.90	0.413	12.13	9.83–15.77	0.545	5.47	4.20–6.50	0.050
	Lung-only metastasis	17.33	15.33–52.57	0.582	14.70	14.00–30.43	0.581	5.47	2.80–7.47	0.680	17.30	15.33–33.30	0.413	14.70	14.00–17.10	0.443	2.87	2.63–10.60	0.720
	Other metastases	23.97	10.77–23.97	0.863	11.23	1.63–29.45	0.154	12.73	9.13–28.70	0.239	16.13	13.55–18.27	0.538	NA	NA	NA	12.13	10.24–15.27	0.532
	Local + distant metastasis	24.87	8.50–34.87	0.247	7.20	3.67–11.57	0.115	5.60	1.73–7.60	0.025	24.11	10.13–30.22	0.094	4.17	2.63–11.57	0.078	6.53	2.10–8.67	0.089
	Multiple metastases	17.50	11.20–19.50	0.006	9.20	8.10–12.63	0.023	5.70	3.80–8.30	0.017	17.50	13.80–19.50	0.049	9.20	8.10–12.63	0.121	5.70	2.70–8.63	0.023
Liver-only metastasis	Reference	20.1	17.37–24.63		12.60	9.83–15.77		5.03	4.07–6.47		20.40	17.37–27.90		12.13	9.83–15.77		5.47	4.20–6.50	
	Lung-only metastasis	17.33	15.33–52.57	0.92	14.70	14.00–30.43	0.676	5.47	2.80–7.47	0.285	17.30	15.33–33.30	0.627	14.70	14.00–17.10	0.752	2.87	2.63–10.60	0.496
	Other metastases	23.97	10.77–23.97	0.562	11.23	1.63–29.45	0.225	12.73	9.13–28.70	0.036	16.13	13.55–18.27	0.452	NA	NA	NA	12.13	10.24–15.27	0.250
	Local + distant metastasis	24.87	8.50–34.87	0.47	7.20	3.67–11.57	0.286	5.60	1.73–7.60	0.931	24.11	10.13–30.22	0.479	4.17	2.63–11.57	0.171	6.53	2.10–8.67	0.773
	Multiple metastases	17.50	11.20–19.50	0.082	9.20	8.10–12.63	0.092	5.70	3.80–8.30	0.076	17.50	13.80–19.50	0.149	9.20	8.10–12.63	0.240	5.70	2.70–8.63	0.855
Lung-only metastasis	Reference	17.33	15.33–52.57		14.70	14.00–30.43		5.47	2.80–7.47		17.30	15.33–33.30		14.70	14.00–17.10		2.87	2.63–10.60	
	Other metastases	23.97	10.77–23.97	0.678	11.23	1.63–29.45	0.125	12.73	9.13–28.70	0.309	16.13	13.55–18.27	0.558	NA	NA	NA	12.13	10.24–15.27	0.326
	Local + distant metastasis	24.87	8.50–34.87	0.491	7.20	3.67–11.57	0.436	5.60	1.73–7.60	0.061	24.11	10.13–30.22	0.553	4.17	2.63–11.57	0.408	6.53	2.10–8.67	0.799
	Multiple metastases	17.50	11.20–19.50	0.275	9.20	8.10–12.63	0.022	5.70	3.80–8.30	0.577	17.50	13.80–19.50	0.718	9.20	8.10–12.63	0.025	5.70	2.70–8.63	0.734
Other metastases	Reference	23.97	10.77–23.97		11.23	1.63–29.45		12.73	9.13–28.70		16.13	13.55–18.27		NA	NA		12.13	10.24–15.27	
	Local + distant metastasis	24.87	8.50–34.87	0.334	7.20	3.67–11.57	0.686	5.60	1.73–7.60	0.016	24.11	10.13–30.22	0.273	4.17	2.63–11.57	NA	6.53	2.10–8.67	0.223
	Multiple metastases	17.50	11.20–19.50	0.108	9.20	8.10–12.63	0.998	5.70	3.80–8.30	0.002	17.50	13.80–19.50	0.617	9.20	8.10–12.63	NA	5.70	2.70–8.63	0.030
Local + distant metastasis	Reference	24.87	8.50–34.87		7.20	3.67–11.57		5.60	1.73–7.60		24.11	10.13–30.22		4.17	2.63–11.57		6.53	2.10–8.67	
	Multiple metastases	17.50	11.20–19.50	0.701	9.20	8.10–12.63	0.958	5.70	3.80–8.30	0.910	17.50	13.80–19.50	0.879	9.20	8.10–12.63	0.770	5.70	2.70–8.63	0.637

NA, not available; other abbreviations as in Table 1.

**Table 3:** Independent prognostic factors for OS.

Characteristics		Head						Body/Tail					
		Univariate analysis			Multivariate analysis			Univariate analysis			Multivariate analysis		
		HR	95%CI	*P*	HR	95%	*P*	HR	95%CI	*P*	HR	95%CI	*P*
Age	≤60 years	Reference		0.126			NI	Reference		0.544			NI
	>60 years	1.388	0.912–2.111					1.336	0.524–3.409				
Gender	Female	Reference		0.271			NI	Reference		0.303			NI
	Male	0.790	0.519–1.202					1.670	0.630–4.428				
WBC	≤10	Reference		0.001	Reference		0.066	Reference		0.648			NI
	>10	2.762	1.553–4.909		0.400	0.151–1.061		0.046	0.000–1.864				
NLR	≤3.32	Reference		0.280			NI	Reference		0.941			NI
	>3.32	1.262	0.827–1.925					0.945	0.210–4.241				
dNLR	≤3.32	Reference		0.457			NI	Reference		0.326			NI
	>3.32	1.193	0.749–1.900					0.630	0.250–1.586				
PLR	≤98.13	Reference		0.274			NI	Reference		0.944			NI
	>98.13	1.657	0.671–4.092					1.034	0.409–2.613				
PNI	0	Reference		0.588			NI	Reference		0.237			NI
	1	1.147	0.699–1.881					3.394	0.449–25.680				
SII	≤1000	Reference		0.838			NI	Reference		0.367			NI
	>1000	1.047	0.677–1.618					0.041	0.000–43.052				
mGPS	0	Reference					NI	Reference					NI
	1	0.964	0.524–1.775	0.907				0.579	0.071–4.739	0.610			
	2	1.353	0.681–2.685	0.338				1.187	0.118–11.974	0.884			
PI	0	Reference			Reference		0.564	Reference					NI
	1	0.393	0.204–0.758	0.005	1.540	0.355–6.684		1.841	0.734–3.123	0.950			
	2	0.460	0.229–0.926	0.030	0.957	0.239–3.826	0.951	1.452	0.665–2.213	0.945			
ALB (g/L)	≤35	Reference		0.838			NI	Reference		0.688			NI
	>35	0.947	0.563–1.594					0.652	0.081–5.244				
CRP (ng/L)	≤3	Reference		0.315			NI	Reference		0.253			NI
	>3	1.244	0.813–1.904					1.964	0.618–6.243				
CA19-9 (U/ml)	≤35	Reference		0.019	Reference		0.340	Reference		0.063			NI
	>35	2.026	1.123–3.656		1.374	0.715–2.638		4.264	0.813–7.831				
CEA (ng/ml)	≤5	Reference		0.103			NI	Reference		0.153			NI
	>5	1.448	0.928–2.261					1.951	0.781–4.873				
HBV infection	Absence	Reference		0.713			NI	Reference		0.573			NI
	Presence	1.186	0.479–2.935					1.801	0.232–13.955				
Chemotherapy	No	Reference		0.240			NI	Reference		0.886			NI
	Yes	0.776	0.509–1.185					0.936	0.376–2.325				
Hemorrhage	Absence	Reference		0.954			NI	Reference		0.402			NI
	Presence	1.043	0.255–4.256					0.038	0–80.925				
Pancreatic fistula	Absence	Reference		0.351			NI	Reference		0.059			NI
	Presence	1.248	0.784–1.986					1.138	0.018–1.082				
Biliary fistula	Absence	Reference		0.702			NI	Reference		0.330			NI
	Presence	1.109	0.652–1.887					0.527	0.145–1.914				
Abdominal infection	Absence	Reference		0.143			NI	Reference		0.887			NI
	Presence	1.553	0.861–2.801					1.160	0.151–8.904				
Time period to recurrence (month)	>24	Reference						Reference			Reference		
	≤6	5.165	2.358–11.316	<0.001	18.337	6.998–48.047	<0.001	10.741	1.340–86.093	0.025	19.452	9.010–53.421	<0.001
	6–12	4.212	1.871–9.484	0.001	13.994	5.341–36.626	<0.001	7.193	0.756–68.448	0.086	5.332	3.761–22.191	<0.001
	12–24	3.072	1.268–7.438	0.013	4.842	1.814–12.923	0.002	11.793	1.140–12.998	0.038	1.391	1.121–3.321	0.011
LN16	Absence	Reference		LA	Reference		0.046	Reference		LA	Reference		0.908
	Presence				2.506	1.017–6.172					2.531	0.932–4.663	
Tumor differentiation	Well	Reference		LA	Reference			Reference		LA	Reference		
	Moderate				2.131	1.252–3.641	0.006				0.011	0.000–0.871	0.043
	Poor				3.522	1.446–5.312	0.002				7.047	1.123–4.196	0.037
Imaging tumor size (cm)	≤2	Reference		LA	Reference			Reference		LA	Reference		
	2–4				0.915	0.417–2.007	0.824				0.209	0.042–2.124	0.985
	>4				1.001	0.492–2.035	0.999				0.180	0.015–2.180	0.178
Local progression	Absence	Reference		LA	Reference		<0.001	Reference		LA	Reference		<0.001
	Presence				7.091	3.645–13.899					0.001	0–0.051	
Liver-only metastasis	Absence	Reference		LA	Reference		<0.001	Reference		LA	Reference		0.001
	Presence				11.490	5.351–24.397					0.012	0.001–0.178	
Lung-only metastasis	Absence	Reference		LA	Reference		0.001	Reference		LA	Reference		0.287
	Presence				4.780	1.871–12.351					0.245	0.018–3.268	
Local + distant metastasis	Absence	Reference		LA	Reference		0.031	Reference		LA	Reference		0.917
	Presence				4.214	1.142–15.550					0.312	0.087–3.042	

NI, not included; LA, included in LASSO analysis. Abbreviations as in Table 1.

**Table 4:** Independent prognostic factors for PFS.

Characteristics		Head						Body/Tail					
		Univariate analysis			Multivariate analysis			Univariate analysis			Multivariate analysis		
		HR	95% CI	*P*	HR	95% CI	*P*	HR	95% CI	*P*	HR	95% CI	*P*
Age	≤60 years	Reference		0.744	Reference		NI	Reference		0.986	Reference		NI
	>60 years	1.051	0.746–1.481					1.006	0.516–1.963				
Gender	Female	Reference		0.525	Reference		NI	Reference		0.537	Reference		NI
	Male	1.120	0.790–1.587					1.232	0.636–2.387				
WBC	≤10	Reference		0.011	Reference		0.393	Reference		0.864	Reference		NI
	>10	1.965	1.165–3.315		1.855	0.450–7.656		1.191	0.161–8.825				
NLR	≤3.32	Reference		0.335	Reference		NI	Reference		0.005	Reference		0.015
	>3.32	1.184	0.840–1.668					3.324	1.441–7.669		3.338	1.267–8.798	
dNLR	≤3.32	Reference		0.815	Reference		NI	Reference		0.858	Reference		NI
	>3.32	1.045	0.723–1.509					0.942	0.492–1.804				
PLR	≤98.13	Reference		0.05	Reference		0.814	Reference		0.255	Reference		NI
	>98.13	2.272	1.001–5.158		1.112	0.460–2.685		1.501	0.746–3.018				
PNI	0	Reference		0.329	Reference		NI	Reference		0.79	Reference		NI
	1	1.232	0.810–1.872					1.137	0.440–2.936				
SII	≤1000	Reference		0.837	Reference		NI	Reference		0.106	Reference		NI
	>1000	1.037	0.732–1.468					2.229	0.843–5.898				
mGPS	0	Reference			Reference		NI	Reference			Reference		
	1	1.122	0.895–1.406	0.319				0.675	0.157–2.911	0.598	1.960	0.212–18.154	0.553
	2	2.11	0.644–1.889	0.214				3.251	0.592–17.861	0.175	13.645	1.175–158.459	0.037
PI	0	Reference			Reference			Reference			Reference		
	1	1.346	1.038–1.745	0.025	0.947	0.198–4.531	0.946	0.708	0.094–5.308	0.737	0.230	0.010–5.382	0.361
	2	1.224	0.987–2.114	0.066	1.496	0.327–6.844	0.604	2.479	0.293–21.006	0.405	0.546	0.117–3.574	0.257
ALB (g/L)	≤35	Reference		0.815	Reference		NI	Reference		0.74	Reference		NI
	>35	0.949	0.614–1.467					0.783	0.184–3.329				
CRP (ng/L)	≤3	Reference		0.138	Reference		NI	Reference		0.018	Reference		0.425
	>3	1.296	0.920–1.825					2.869	1.197–6.877		1.579	0.849–4.552	
CA19-9 (U/ml)	≤35	Reference		0.020	Reference		0.364	Reference		0.086	Reference		NI
	>35	1.762	1.094–2.838		0.774	0.445–1.346		2.347	0.885–6.225				
CEA (ng/ml)	≤5	Reference		0.010	Reference		0.007	Reference		0.320	Reference		NI
	>5	1.595	1.118–2.274		1.785	1.167–2.730		1.392	0.725–2.675				
HBV infection	Absence	Reference		0.997	Reference		NI	Reference		0.331	Reference		NI
	Presence	0.999	0.507–1.966					2.735	0.360–20.747				
Chemotherapy	No	Reference		0.054	Reference		0.001	Reference		0.690	Reference		NI
	Yes	1.396	0.994–1.961		0.476	0.302–0.749		1.141	0.594–2.195				
Hemorrhage	Absence	Reference		0.990	Reference		NI	Reference		0.585	Reference		NI
	Presence	1.008	0.320–3.170					0.570	0.076–4.269				
Pancreatic fistula	Absence	Reference		0.407	Reference		NI	Reference		0.237	Reference		NI
	Presence	1.178	0.800–1.733					0.549	0.203–1.482				
Biliary fistula	Absence	Reference		0.971	Reference		NI	Reference		0.873	Reference		NI
	Presence	0.991	0.627–1.568					1.071	0.460–2.495				
Abdominal infection	Absence	Reference		0.313	Reference		NI	Reference		0.348	Reference		NI
	Presence	1.319	0.770–2.257					2.641	0.348–20.038				
LN16	Absence	Reference		LA	Reference		0.123	Reference		LA	Reference		NI
	Presence				1.939	0.835–4.503							
Tumor differentiation	Well	Reference		LA	Reference		NI	Reference		LA	Reference		NI
	Moderate												
	Poor												
Imaging tumor size (cm)	≤2	Reference		LA	Reference			Reference		LA	Reference		NI
	2–4				0.974	0.425–2.231	0.951						
	>4				1.703	1.195–3.648	0.029						
Local progression	Absence	Reference		LA	Reference		<0.001	Reference		LA	Reference		NI
	Presence				13.643	7.279–25.569							
Liver-only metastasis	Absence	Reference		LA	Reference		<0.001	Reference		LA	Reference		NI
	Presence				18.632	10.506–33.043							
Lung-only metastasis	Absence	Reference		LA	Reference		<0.001	Reference		LA	Reference		NI
	Presence				19.313	7.054–52.877							
Local + distant metastasis	Absence	Reference		LA	Reference			Reference		LA	Reference		NI
	Presence				13.535	5.905–31.024	<0.001						
Multiple metastases	Absence	Reference		LA	Reference		NI	Reference		LA	Reference		NI
	Presence				33.96	13.137–87.808							
TNM stage	IA	Reference		LA	Reference			Reference		LA	Reference		NI
	IB				1.382	0.476–4.009	0.552						
	IIA				1.253	0.486–3.232	0.64						
	IIB				4.401	1.537–12.602	0.006						
	III				1.776	0.734–4.299	0.203						
Back membrane invasion	Absence	Reference		LA	Reference		0.09	Reference		LA	Reference		NI
	Presence				1.460	0.943–2.262							
LNR	0	Reference		LA	Reference		NI	Reference		LA	Reference		NI
	0–0.16				0.434	0.144–1.306	0.137						
	>0.16				1.043	0.576–1.886	0.890						
Positive LN number	0	Reference		LA	Reference			Reference		LA	Reference		NI
	1–3				0.416	0.144–1.203	0.106						
	>4				0.587	0.274–1.587	0.323						
Pathological size (cm)	≤2	Reference		LA	Reference		NI	Reference		LA	Reference		
	2–4										0.099	0.016–0.625	0.014
	>4										0.556	0.252–1.227	0.146
Imaging LN size (cm)	≤0.5	Reference		LA	Reference		NI	Reference		LA	Reference		
	0.5–1.5										0.254	0.086–0.751	0.013
	>1.5										2.999	0.813–11.060	0.099
Imaging vascular invasion	Absence	Reference		LA	Reference		NI	Reference		LA	Reference		0.239
	Presence										1.670	0.712–3.919	

NI, not included; LA, Included in LASSO analysis. Abbreviations as in Table 1.

**Table 5:** Comparison of the C-index and AUC values between predictive systems and TNM stage.

System	Head										Body/tail									
	OS					PFS					OS					PFS				
	C-index	AUC			*P*	C-index	AUC			*P*	C-index	AUC			*P*	C-index	AUC			*P*
		1-year	2-year	3-year			1-year	2-year	3-year			1-year	2-year	3-year			1-year	2-year	3-year	
Predictive system	0.688 (0.623–0.753)	0.720	0.734	0.801	0.004	0.800 (0.760–0.840)	0.749	0.749	0.748	0.001	0.751 (0.611–0.891)	0.684	0.688	0.672	0.050	0.802 (0.736–0.868)	0.848	0.740	0.741	0.001
8th TNM stage	0.600 (0.536–0.664)	0.598	0.580	0.601		0.616 (0.565–0.667)	0.612	0.650	0.619		0.633 (0.500–0.768)	0.538	0.548	0.520		0.671 (0.571–0.771)	0.572	0.540	0.597	

C-index, concordance index; AUC, area under receiver operating characteristic curves.

## Data Availability

The authenticity of this article has been validated by uploading the key raw data onto the Research Data Deposit public platform (http://www.researchdata.org.cn), with the approval number as RDDA2020001531.
